# Metastasis of colon cancer to the thyroid and cervical lymph nodes: a case report

**DOI:** 10.1186/s40792-016-0237-3

**Published:** 2016-10-07

**Authors:** Shigeki Minami, Keiji Inoue, Junji Irie, Takashi Mine, Nobuhiro Tada, Masataka Hirabaru, Kazumasa Noda, Shinichiro Ito, Masashi Haraguchi

**Affiliations:** 1 Department of Surgery, Nagasaki Harbor Medical Center City Hospital, 6-39 shinchi-machi, Nagasaki, 850-8555 Japan; 2 Department of Pathology, Nagasaki Harbor Medical Center City Hospital, 6-39 shinchi-machi, Nagasaki, 850-8555 Japan; 3 Department of Clinical Oncology, Nagasaki Harbor Medical Center City Hospital, 6-39 shinchi-machi, Nagasaki, 850-8555 Japan

**Keywords:** Thyroid, Metastasis, Colon cancer

## Abstract

The incidence of thyroid metastasis among colorectal cancer patients is extremely rare. We report a case of colonic adenocarcinoma metastasis to the thyroid gland with treatment of lung and liver metastases, in a 61-year-old woman with a history of colon cancer. She showed a thyroid mass related to a 3-month history of hoarseness. Physical and imaging examinations disclosed a diffuse large thyroid mass with swollen cervical lymph nodes. Fine-needle aspiration cytology of the thyroid mass suggested malignancy. The patient underwent total thyroidectomy. Histopathological examination and immunohistochemical staining revealed adenocarcinoma, which was consistent with a diagnosis of metastases from primary colon cancer to the thyroid and cervical lymph nodes. At 2 years after thyroid surgery, the patient has been continuing outpatient chemotherapy for the lung and liver metastases. Thyroidectomy appeared to both relieve the patient and prevent local symptoms.

## Background

Clinically evident metastatic disease to the thyroid gland is rare. Metastases from non-thyroid malignancies to the thyroid gland have been reported in 1.4 to 3 % of all patients who have surgery for suspected cancer in the thyroid gland [1]. The incidence among colorectal cancer patients is rare. We, herein, report the case of a patient with a history of colon cancer which metastasized to the thyroid. Approval from our hospital's Institutional Review Board was obtained for this case report.

## Case presentation

A 61-year-old woman with a 3-month history of hoarseness was referred to our department for evaluation of a thyroid mass. Her family history was noncontributory. Five years previously, she had undergone an excision of the sigmoid colon for well-differentiated adenocarcinoma of the sigmoid colon (pSS, pN0, M0 stage II). At 1 year and 8 months after the surgery, a computed tomography (CT) scan performed as part of a postoperative follow-up study revealed a metastasis in S10 of the left lung. The patient underwent a left lung basal segmentectomy. Postoperatively, she received chemotherapy with modified FOLFOX6. At 1 year and 3 months after the second surgery, a CT scan detected asynchronous multiple lung metastases in S6 of the right lung, S3 and S5 of the left lung, and the left lower lobe. At 6 months after the lung metastasis detection, she underwent a thoracoscopic lung surgery of partial resection of S6 of the right lung and S3 and S5 of the left lung and a left lung lower lobectomy. After these procedures, she received oral chemotherapy with S-1. At 10 months after the third surgery, she underwent a laparoscopic partial hepatectomy for liver metastasis in S5 of the liver.

At 7 months after liver surgery, she noticed a swelling in the front of her neck and gradually felt increasing hoarseness. Physical examination revealed a hard diffuse goiter, 8 × 6 cm in size. Also, hard enlarged lymph nodes were palpated in the cervical lymph node region. The laboratory data revealed no anemia and no abnormalities in the liver, renal, or thyroid function, but serum levels of carcinoembryonic antigen and thyroglobulin were found to be elevated at 9.8 ng/ml (normal 0.0 to 5.5 ng/ml) and 176.8 ng/ml (0.0 to 32.7 ng/ml), respectively.

**Fig. 1:**
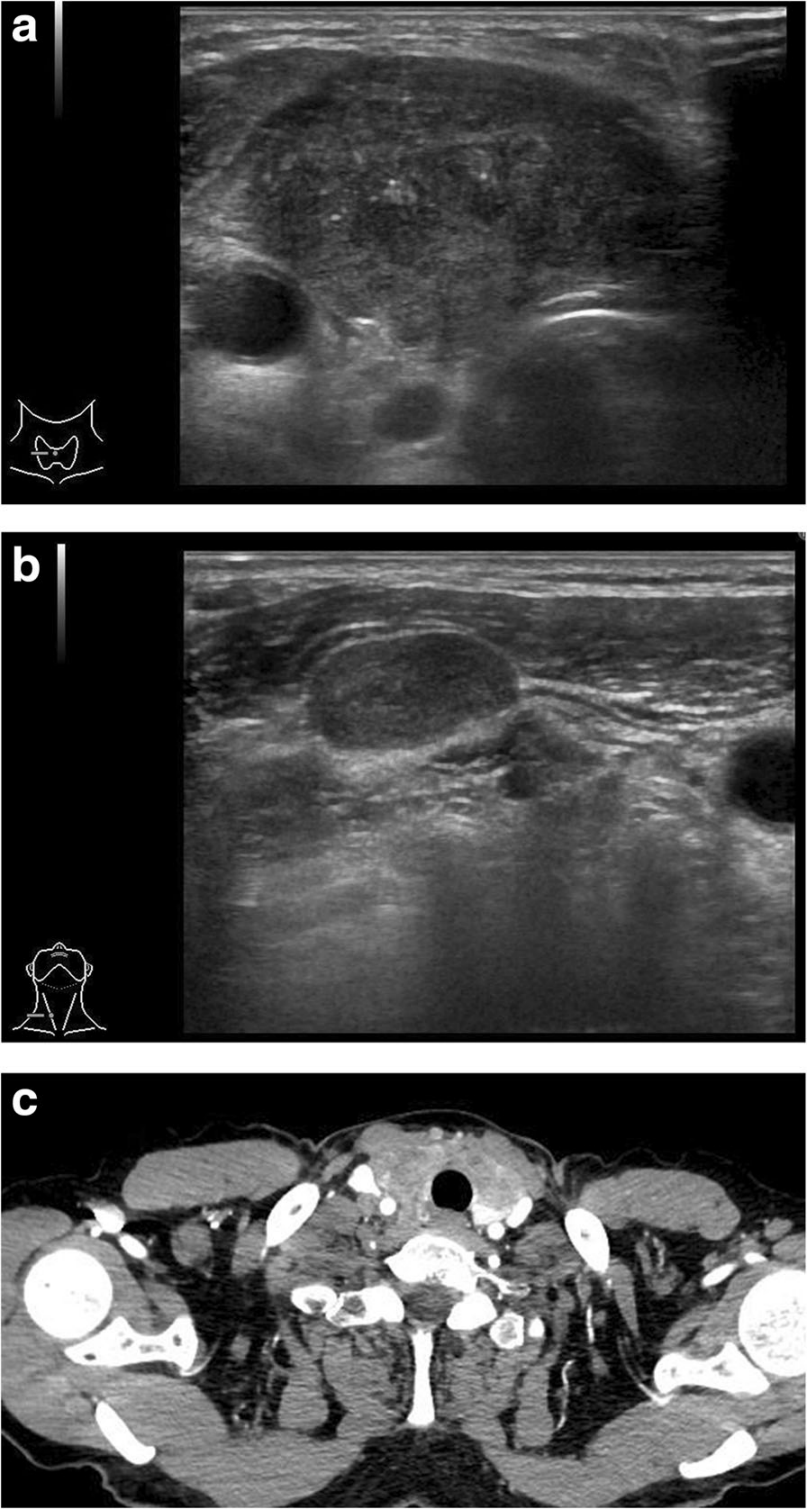
Ultrasound and CT scan findings. **a** Ultrasonography shows a hypoechoic mass in the thyroid. **b** Swollen bilateral cervical lymph nodes. **c** Low-density mass is spread in the thyroid with no evidence of invasion of the trachea

Ultrasonography disclosed a hypoechoic mass in the thyroid and swollen bilateral cervical lymph nodes (Fig. 1). CT scans revealed a low-density widespread mass in the thyroid and swollen bilateral cervical lymph nodes. There was no evidence of invasion of the trachea. At this time, chest CTs showed several small lung metastases and a single metastasis of the liver at S8. The patient underwent fine-needle aspiration cytology (FNAC) of the thyroid tumor. FNAC showed tumor cell clusters that were suspected to be thyroid papillary carcinoma, suggesting malignancy.

**Fig. 2:**
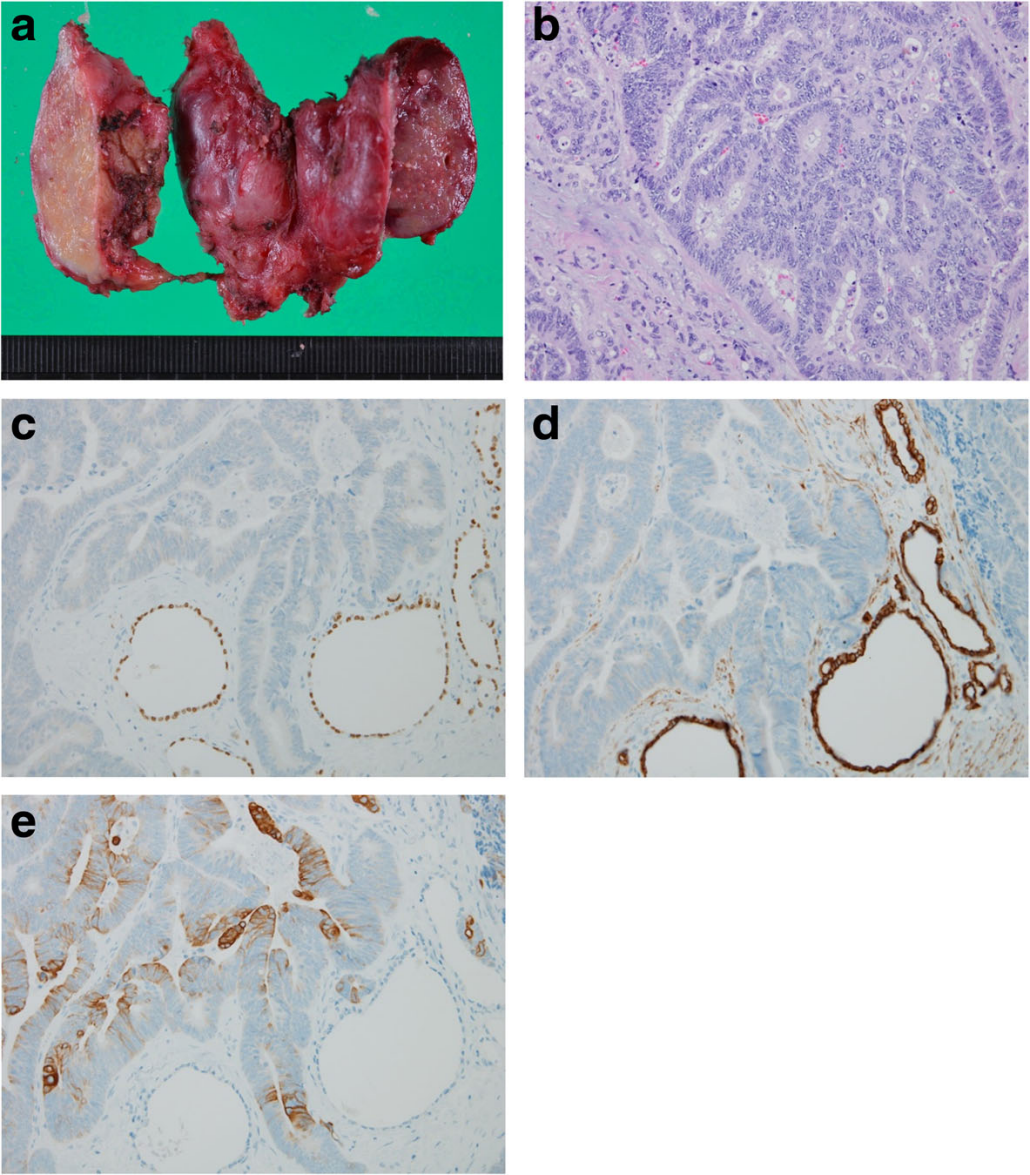
Histopathological and immunohistochemical (IHC) staining findings of the thyroid. **a** Gross findings reveal an ill-defined white diffuse mass. **b** Microscopic findings show cribriform nests of tall columnar cells accompanied by necrosis (H&E stain, ×400). **c** IHC staining for TTF-1 reveals negative staining in the tumor cells. **d** IHC staining for cytokeratin 7 shows negative staining in the tumor cells. **e** IHC staining for cytokeratin 20 shows cytoplasmic positivity in the tumor cells

**Fig. 3:**
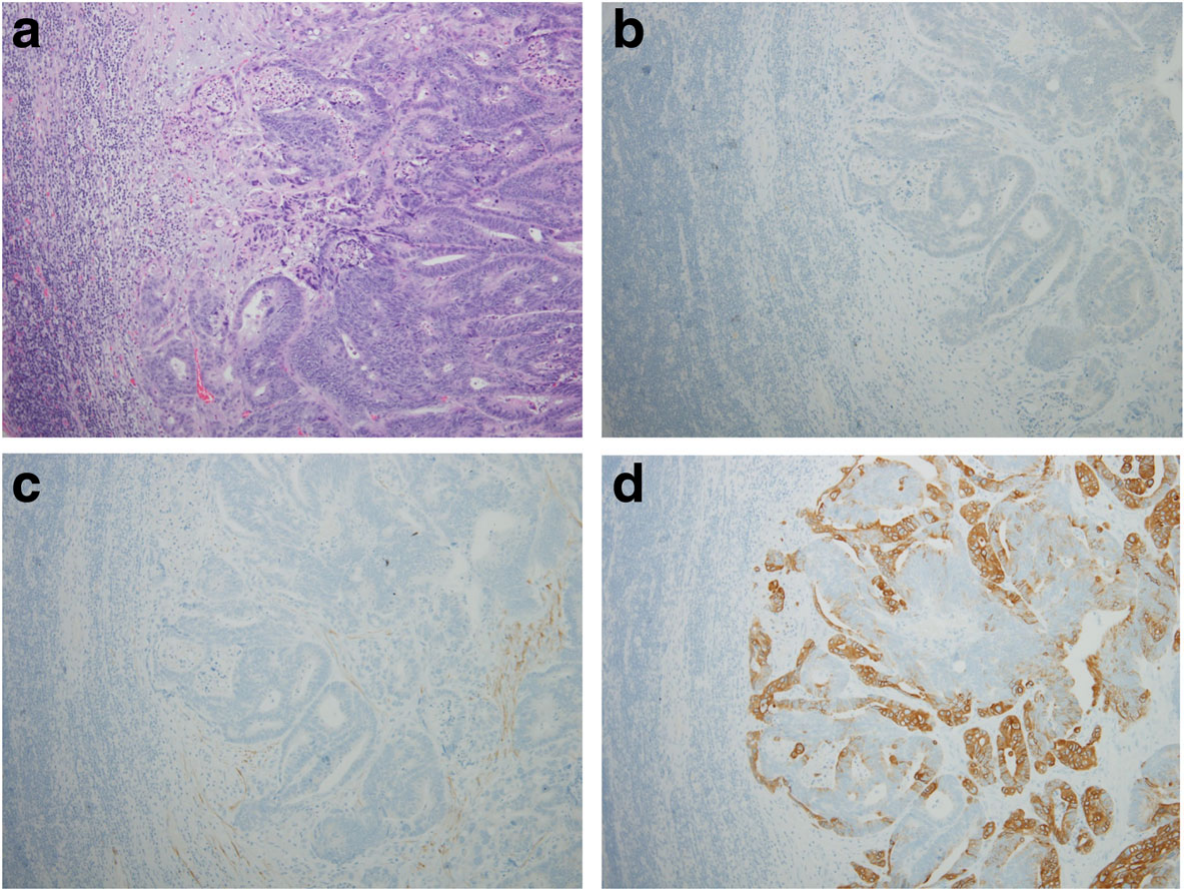
Histopathological and immunohistochemical (IHC) staining findings of the cervical lymph nodes. **a** Microscopic findings show cribriform nests of tall columnar cells in one of the affected lymph nodes (H&E stain, ×400). **b** IHC staining for TTF-1 reveals negative staining in the tumor cells. **c** IHC staining for cytokeratin 7 shows negative staining in the tumor cells. **d** IHC staining for cytokeratin 20 shows cytoplasmic positivity in the tumor cells

Subsequently, the patient underwent a total thyroidectomy and bilateral cervical lymph nodes dissection. Intraoperatively, the tumor was found to involve the right recurrent laryngeal nerve, which had to be sacrificed. Histopathological examination showed cribriform nests of tall columnar cells accompanied by necrosis. These results suggested adenocarcinoma, not papillary carcinoma. The lesion occupied almost the entirety of the thyroid (Fig. 1). Next, immunohistochemical (IHC) staining was performed using cytokeratin 7 (CK7), cytokeratin 20 (CK20), and thyroid transcription factor-1 (TTF-1), in order to clarify the origin of the tumor. The tumor cell test results were positive for CK20 and negative for CK7 and TTF-1 (Fig. 2). Histopathological and IHC examinations of the cervical lymph nodes metastases showed the same results as in the thyroid tumor (Fig. 3). These findings were consistent with colon cancer metastasis, although we did note that the cervical lymph nodes metastases were similar to those in thyroid cancer cases. Therefore, it appeared clear that the thyroid tumor was a metastasis from the colon carcinoma. The patient was discharged from our hospital on the seventh postoperative day. At 2 years after thyroid surgery, the patient has been continuing outpatient chemotherapy for the lung and liver metastases.

### Discussion

Metastases to the thyroid gland are uncommon. The incidence of thyroid metastasis among colorectal cancer patients is quite low: this diagnosis is applied to 6 patients (0.1 %) out of a total of 5862 colorectal cancer patients [1]. Recently, Chung et al. [2] reviewed all clinical reports available in the literature from 2000 to 2010 including case reports and case series. In this analysis of thyroid metastases, primary cancers were identified as renal (48.0 % of cases), colorectal (10.4 %), lung (8.3 %), breast (7.8 %), or sarcoma (4.0 %).

While 60 to 80 % of metastases are metachronous (following previously treated malignancy), 20 to 40 % are synchronous (simultaneous) with the primary lesion [3]. In metachronous metastases, the mean and median intervals between diagnosing primary malignancies and their metastases to the thyroid gland were reported at 69.9 and 53 months, respectively [2]. The mean interval between discovery of primary tumor and of thyroid metastasis was 68 months for renal cancer, 48.2 months for breast cancer, 41.5 months for colorectal cancer, and 20.9 months for malignant melanoma. Another report indicated a very poor prognosis for thyroid metastasis from colorectal cancer: a cancer-related death rate of 50 % in less than a year [3]. For such cases, systemic management depending on the primary colon cancer may contribute to longer survival following thyroid surgery.

Froylich et al. [4] reviewed metachronous colon metastasis to the thyroid. The authors found 34 cases; two thirds of the patients were female and patient age ranged from 34 to 85. The metastases’ primary sites were the rectum (41 %), sigmoid colon (33 %), right colon (19 %), or left colon (11 %). The staging of the colon carcinoma was stage III or IV in 75 % of the patients. Metastasis to the thyroid was diagnosed 6 months to 8 years after colonic resection. In our case, the staging of the sigmoid colon cancer was stage II. The thyroid metastasis was revealed at 5 years after sigmoid colon cancer surgery.

Although a high sensitivity has been reported for FNA in patients with various metastatic carcinomas to the thyroid gland, the accuracy of FNA is around 50 % [5, 6]. IHC is usually able to differentiate between primary thyroid malignancy and secondary malignancy [7, 8]. In general, thyroid tumor test results are positive for CK7 and negative for CK20. In contrast, colon cancer results are almost always positive for CK20 and negative for CK7. In our case, the tumor cell results were positive for CK20 and negative for CK7.

The role of surgery for treating thyroid metastasis is unclear. Thyroidectomy constitutes better palliation of respiratory symptoms than mere observation [9]. Local control of metastatic disease in the central compartment of the neck can be successfully achieved with minimal morbidity with surgical resection in selected patients [5]. Nonsurgical treatments, such as radiotherapy and chemotherapy, have been used for patients deemed inoperable, although the impact of these modalities remains uncertain [10]. Although our patient had recurrent laryngeal nerve palsy, surgery appeared to give her relief and prevention from local symptoms.

## Conclusions

In conclusion, thyroidectomy in a thyroid metastasis patient appeared to both relieve the patient and prevent the occurrence of local symptoms.
